# HAHmiR.DB: a server platform for high-altitude human miRNA–gene coregulatory networks and associated regulatory circuits

**DOI:** 10.1093/database/baaa101

**Published:** 2020-12-01

**Authors:** Pankaj Khurana, Apoorv Gupta, Ragumani Sugadev, Yogendra Kumar Sharma, Bhuvnesh Kumar

**Affiliations:** Defence Institute of Physiology and Allied Sciences (DIPAS), Defence R&D Organization (DRDO), Lucknow Road, Timarpur, Delhi 110054, India; Defence Institute of Physiology and Allied Sciences (DIPAS), Defence R&D Organization (DRDO), Lucknow Road, Timarpur, Delhi 110054, India; Defence Institute of Physiology and Allied Sciences (DIPAS), Defence R&D Organization (DRDO), Lucknow Road, Timarpur, Delhi 110054, India; Defence Institute of Physiology and Allied Sciences (DIPAS), Defence R&D Organization (DRDO), Lucknow Road, Timarpur, Delhi 110054, India; Defence Institute of Physiology and Allied Sciences (DIPAS), Defence R&D Organization (DRDO), Lucknow Road, Timarpur, Delhi 110054, India

## Abstract

Around 140 million people live in high-altitude (HA) conditions! and even a larger number visit such places for tourism, adventure-seeking or sports training. Rapid ascent to HA can cause severe damage to the body organs and may lead to many fatal disorders. During induction to HA, human body undergoes various physiological, biochemical, hematological and molecular changes to adapt to the extreme environmental conditions. Several literature references hint that gene-expression-regulation and regulatory molecules like miRNAs and transcription factors (TFs) control adaptive responses during HA stress. These biomolecules are known to interact in a complex combinatorial manner to fine-tune the gene expression and help in controlling the molecular responses during this stress and ultimately help in acclimatization. High-Altitude Human miRNA Database (HAHmiR.DB) is a unique, comprehensive and curated collection of miRNAs that have been experimentally validated to be associated with HA stress, their level of expression in different altitudes, fold change, experiment duration, biomarker association, disease and drug association, tissue-specific expression level, Gene Ontology (GO) and Kyoto Encyclopaedia of Gene and Genomes (KEGG) pathway associations. As a server platform, it also uniquely constructs and analyses interactive miRNA–TF–gene coregulatory networks and extracts regulatory circuits/feed-forward loops (FFLs). These regulatory circuits help to offer mechanistic insights into complex regulatory mechanisms during HA stress. The server can also build these regulatory networks between two and more miRNAs of the database and also identify the regulatory circuits from this network. Hence, HAHmiR.DB is the first-of-its-kind database in HA research, which is a reliable platform to explore, compare, analyse and retrieve miRNAs associated with HA stress, their coregulatory networks and FFL regulatory-circuits. HAHmiR.DB is freely accessible at http://www.hahmirdb.in

## Introduction

High altitude (HA) is defined as a height between 2000 and 4000 m above sea level; very high altitude is between 4000 and 5500 m, and altitudes >5500 m are considered extremely high altitude. These altitudes affect the normal physiology and health due to the low partial pressure of oxygen at these altitudes (1). Oxygen concentration and the barometric pressure at sea level is about 21% and 760 mmHg, respectively, but the barometric pressure decreases gradually and significantly at higher altitudes, which also affects the oxygen concentration. At an altitude of 3500 m, there are 40% fewer oxygen molecules per breath (1). Hence, the supply of oxygen to the body tissues also decreases significantly. This condition is known as hypobaric hypoxia (2). To oxygenate the body adequately, the hypoxic condition triggers an acclimatization mechanism both at the physiological and molecular levels. At the physiological level, the body hyperventilates to increase the oxygen concentration in the blood. The blood pressure increases to augment the oxygen supply to the tissues (3). But a sudden increase in the blood flow can also cause the fluid to leak from the blood capillaries, and this fluid build-up in the lungs and the brain triggers life-threatening illnesses, i.e. high-altitude pulmonary oedema (HAPE) and high-altitude cerebral oedema (HACE) (4). At the molecular level, the lack of oxygen (hypoxia) is sensed by the oxygen sensor of cells called prolyl hydroxylases (PHDs) (3). These PHDs further activate the master transcription factor (TF) of hypoxia known as hypoxia-inducible factor‐1 (HIF‐1) (5), which largely controls the signalling machinery responsible for hypoxia-adaptive responses in the body (3). These molecular responses in hypoxia adaptation are controlled by the fine-tuning of the transcriptome expression (6). The gene regulatory elements, such as TFs and miRNAs, are crucial as they have been found to play a decisive role in maintaining physiological and molecular homeostasis, both under normoxic and hypobaric hypoxia conditions (7). miRNAs are known to regulate a number of molecular mechanisms during HA acclimatization or disorders, e.g. miR-16, -20b, -22 and -206 and -17/92 are downregulated during HAPE and are responsible for the disruption of the ion channels and loss of cellular integrity (8). Increase in the red blood cell count, haematocrit values and haemoglobin are established physiological responses at HA. hsa-miR-210-3p (also known as the master regulator hypoxiamiR) expression is positively correlated with the change in red blood cell counts and haemoglobin and haematocrit values at HA and is proposed to be associated with human acclimatization to life at HA (9). Human plasma miRNA expression profiles are thus reported to be associated with human adaptation to hypobaric-hypoxic environments (10). hsa-miR-369-3p, hsa-miR-449b-3p and hsa-miR-136-3p; miR-495 and miR-323a-3p and miR-500a-5p and miR-501-5p have been reported to be differentially expressed at different altitudes and durations of exposure and have been proposed to have immense potential as markers or therapeutic targets (11). miRNAs are known to interact with other regulatory molecules like TFs. The single-nucleotide polymorphism variants of some of the TFs like EPAS1, EGLN1 and PPRAG inherent in the Tibetan population are reported as important ecological traits for HA adaptation (12–14). Several reports hint towards TF and miRNA working in conjunction to influence the precise control and fine-tuning of gene expression during multifactorial disorders like myocardial infarct, cancer, multiple sclerosis and schizophrenia. They are also known to regulate a variety of processes in hypoxia-associated disorders like malignancies, wound repair and stroke (15–20).

A tripartite interaction between an miRNA, TF and a common gene forms regulatory circuits also known as a feed-forward loop (FFL). In FFL, either the TF or the miRNA or both regulate each other and also a target gene. It can be further categorized as miRNA–FFL and TF–FFL. In an miRNA–FFL, miRNA is the main regulator, which regulates a TF and its common target gene, while in a TF–FFL, TF is the main regulator (20). These FFL motifs have been found to play vital roles in disease pathology, drug repurposing and disease recurrence, and hence can be used for the understanding of the underlying mechanism of disease initiation, progression and recurrence (16, 21). Recently, miRNA-based FFLs are proposed as potential biomarkers for complex multifactorial disorders like colorectal cancer and myocardial infarct (21, 22). Analysis of miRNA–TF–gene coregulatory networks in these diseases has found to successfully predict the disease pathology and recurrence.

Recently, there have been a number of studies assessing miRNA expression and regulation during HA stress. This molecular data are scattered, and our study is a systematic attempt to collect, curate, analyse, visualize and store this data. High-Altitude Human miRNA Database (HAHmiR.DB) is a first-ever comprehensive database for miRNA associated with HA stress, their coregulatory networks and regulatory circuits. The database currently contains 384 human miRNAs that are manually curated from peer-reviewed publications related to high-throughput techniques such as microarray, qPCR and RNA seq. For each miRNA, the database stores the experiment details, altitude and duration of experiment, level of regulation (up/down), fold change, Gene Expression Omnibus (GEO) accession, association as biomarker and a corresponding link to the respective publication. The database also collates mature miRNA Ids, miRNA and RNAcentral accession, family, precursor and mature sequence and the stem-loop structure. It also retrieves and stores the miRNA gene targets. Using these, miRNA–TF–gene coregulatory networks are built, and two types of FFLs, i.e. miRNA–FFL and TF–FFL, are extracted using in-house scripts. The database also stores other information about miRNAs like their role in other diseases, its tissue-specific expression and their association with drugs. GO and KEGG pathway enrichment of the miRNA targets are also stored. As a server platform, HAHmiR.DB also builds interactive and dynamic miRNA–TF–gene coregulatory networks of user-defined miRNA list and also identifies the FFL regulatory circuits from the network. HAHmiR.DB will help to uncover the underlying cross-talk between miRNA, TF and gene that governs the fine-tuning of gene expression during HA acclimatization. It will be useful in revealing novel and robust targets and important regulating modules responsible for acclimatizing to HA. Researchers can further use this information for getting mechanistic insights into the complex molecular responses in HA-related disorders.

## Material and methods

### Data collection

A combination of keywords such as ‘High Altitude’ and ‘miRNA’ was used to extract the list of publications from PubMed and Google Scholar as of January 2020. After removing redundancy and duplicity, the miRNA–HA associations were manually curated from the following publications: Yan Yan et al. (168 miRNAs associations) (10), Bao Liu et al. (87 miRNAs associations) (23), Liping Sun et al. (121 miRNAs associations) (24), Si-Qing Ma et al. (39 miRNAs associations) (25), Bao Liu et al. (4 miRNAs associations) (26), He Huang et al. (2 miRNAs associations) (27), Yan Yan et al. (2 miRNAs associations) (9), Birgit Blissenbach et al. (4 miRNA associations) (28), Perwez Alam et al. (60 miRNA associations) (29) and Norman E Buroker et al. (69 miRNA associations) (30). For each miRNA, its experimental group, experiment altitude, duration of induction, level of expression, fold change, GEO accession and its reference paper were compiled. Sometimes, the information, e.g. fold change and the tissue of expression, was not categorically listed in the publication, so those entries are recorded as ‘na’. We also recorded whether any literature text had associated the miRNA as a biomarker for HA acclimatization. Subsequently, miRBase stem-loop ID, miRBase accession number, miRNA family, 5’ ID, 3’ ID, chromosome number, precursor sequence, mature sequence and RNA central ID for each miRNA were retrieved and stored from the mirbase version release 22.1 (31), miRGen version 2.0 (32) and RNAcentral version release 14 (33) databases, respectively. To ensure uniformity in the nomenclature, the names of precursor miRNAs were mapped to the mature miRNA using miRBase version release 22.1 (31) and miRDB (34) databases.

### Data annotation

A comprehensive and exhaustive list of human TFs and Co-TFs were mined from TFcheckpoint, DBD version 2.0, TcoF-DB version 2.0 and TRANSFAC version 7.0 (35–38). This was stored as a human TF list. For each miRNA, its experimentally validated gene targets were identified from MirTarBase version 7.0 and miRecords (last updated on 27 April 2013) (39, 40). These gene targets were annotated as TF or gene by comparison with the human TF list. In this way, the corresponding miRNA→gene interactions were annotated as miRNA→TF or miRNA→gene interactions.

Further TF→gene and TF→miRNA interactions were compiled from OregAnno version 3.0 (41), TRRUST version 2.0 (42) and TransmiR version 2.0 (43), PutmiR version 1.1 (44) databases, respectively. Using these miRNA→gene, miRNA→TF, TF→ ene, TF→miRNA interactions, an miRNA–TF–gene coregulatory network was built for each miRNA in the database. Additionally, miRNA→miRNA interactions in the database were also added from the PmmR (45) database to identify more complex network interactions.

**Figure 1.**
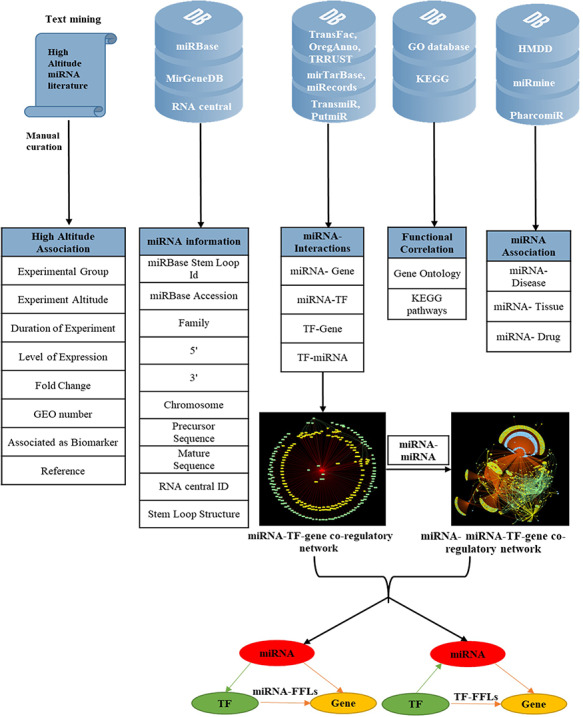
Overview of data collection and annotation in HAHmiR.DB. A comprehensive list of differentially expressed HA-associated human miRNAs was mined from the published literature with its HA-associated information, i.e experimental group, experiment altitude, duration of experiment, level of expression, fold change, GEO accession and association as biomarker. These miRNAs were further enriched with a variety of other information from generic biological databases that includes miRNA sequence–related information, miRNA stem-loop structure, miRNA–network interactions (miRNA–gene, miRNA–TF, TF–miRNA, TF–gene), functional enrichments (Gene Ontology, pathways), miRNA–disease association, miRNA–drug association and miRNA–tissue expression.

To make the database more informative, several other attributes were added; miRNA–disease associations were mined from HMDD version 3.0 (46); miRNA–tissue association from miRmine (47) and miRNA–drug relationship from PharmacomiR (48). For each miRNA, GO-functional enrichment and KEGG pathway enrichment of their target genes were performed using Database for Annotation, Visualization and Integrated Discovery (49). The complete overview of data collection and annotation is listed in Figure 1.

### FFL motif identification

Each vertex in the network is labelled as V_m_, V_TF_ or V_gene_, where V_m_ refers to an miRNA, V_TF_ refers to a TF and V_gene_ refers to a gene. The edges are annotated as E_mt_, E_mg_, E_tg_, E_tm_, where E_mt_ refers to the edge from V_m_ to V_TF_, E_mg_ refers to the edge from V_m_ to V_gene_, E_tg_ refers to the edge from V_TF_ to V_gene_ and E_tm_ refers to the edge from V_TF_ to V_m_.

For each V_m_, its edge E_mg_ from the miRNA→gene interactions is searched in miRNA→TF interaction to identify a corresponding E_mt_ with a common vertex V_m_. Thereafter, an analogous E_tg_ is searched in the TF→gene interactions. If found, the complete graph containing three vertices (V_m_, V_TF_, V_gene_) and edges (E_mt_, E_mg_, E_tg_) were labelled as miRNA–FFL motif graph.

Similarly, in order to find TF–FFL motif, edges E_tm_ and E_tg_ were identified from TF→miRNA and TF→gene interactions data set, respectively. Further, the program scans for an E_mg_ associated with V_m_ and V_gene_ in miRNA→gene interaction data set. If algorithm finds E_mg_, the complete graph containing three vertices (V_m_, V_TF_, V_gene_) and edges (E_tm_, E_mg_, E_tg_) were labelled as TF–FFL motif graph. This methodology was used to identify miRNA–FFLs and TF–FFLs for each human miRNA.

### Database development

Finally, after collection, processing and enrichment of the data, all the database files were stored as JavaScript Object Notation (JSON) files in MongoDB database relational database (50). These JSON files were uploaded on the server localhost using PyMongo, and query commands were made in the command line client in MongoDB compass. The database uses Asynchronous JavaScript and XML (AJAX) technique for API calls. AJAX is a web development technique used for creating interactive web applications. It utilizes XHTML for content along with the document object model and JavaScript for dynamic content display. Vis.js library functions are used for interactive visualization of network graphs on front-end. The website is available online at www.hahmirdb.in and requires no registration. It provides a user-friendly interface to browse and analyse HA-associated miRNAs.

## Results

**Figure 2.**
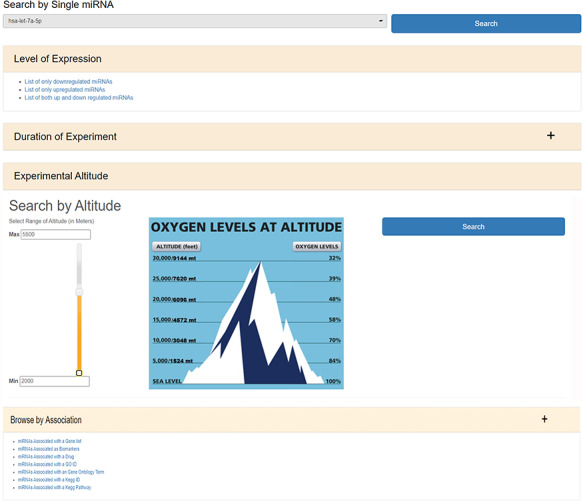
The web image of HAHmiR.DB—‘Browse’ option allows the user to browse differentially expressed (DE) miRNA during HA acclimatization using multiple filters, i.e. level of expression, duration of experiment, experimental altitude and browse by associations that includes filters such as miRNAs Associated with a Gene List, miRNAs Associated as Biomarkers, miRNAs Associated with a Drug, miRNAs Associated with a GO ID/term, miRNAs Associated with a KEGG ID/Pathway.

HAHmiR.DB database allows to browse, retrieve, analyse and compare miRNAs that are associated with HA stress. In ‘Browse’ option, user can either search individual miRNA directly from a pull-down menu or can use a variety of filters to fetch the miRNAs of interest. The ‘Search by Single miRNA’ option allows the user to select a miRNA from a drop-down menu and further link to its detailed information page. The database also provides user with four other filters to select and retrieve the list of miRNAs (Figure 2). These filters are based on the level of expression (upregulated/downregulated/both up- and downregulated); duration of experiment (days/months/native), experimental altitude (2000–7600 m) and by ‘Search by Association’. The experimental altitude option is provided with a slider bar, where user can select the altitude range (2000–7600 m) to get the set of miRNAs associated with that extent. The fourth browse option of the database ‘Browse by Association’ is the special option, where user can retrieve a list of miRNAs associated with gene list/biomarker/drug/GO ID/GO term/KEGG ID/KEGG term.

The ‘miRNAs Associated with a Gene List’ option allows the user to select gene(s) from a pull-down gene list and fetch the miRNAs regulating these genes (Supplementary Figure S1a). The ‘miRNAs Associated as Biomarkers’ option allows the user to extract the list of miRNAs that are associated as biomarkers under HA conditions (Supplementary Figure S1b). Similarly, the ‘miRNAs Associated with a Drug’ option allows the user to identify miRNAs associated with a particular drug (Supplementary Figure S1c). The ‘miRNAs Associated with a GO ID’ (Supplementary Figure S1d), ‘miRNAs Associated with a GO Term’ (Supplementary Figure S1e), ‘miRNAs Associated with a KEGG ID’ (Supplementary Figure S1f) and ‘miRNAs Associated with a KEGG Pathway’ (Supplementary Figure S1g) options allow the user to fetch miRNAs based on functional association such as GO ID, GO term, KEGG ID and KEGG pathway, respectively. All the above options provide a resultant list of miRNAs that are hyperlinked to their respective detailed information pages. The list of these miRNAs can be downloaded in Excel/PDF format for further analysis.

The miRNA information page of HAHmiR.DB can be broadly divided into five sections.

### Knowledge base

**Figure 3.**
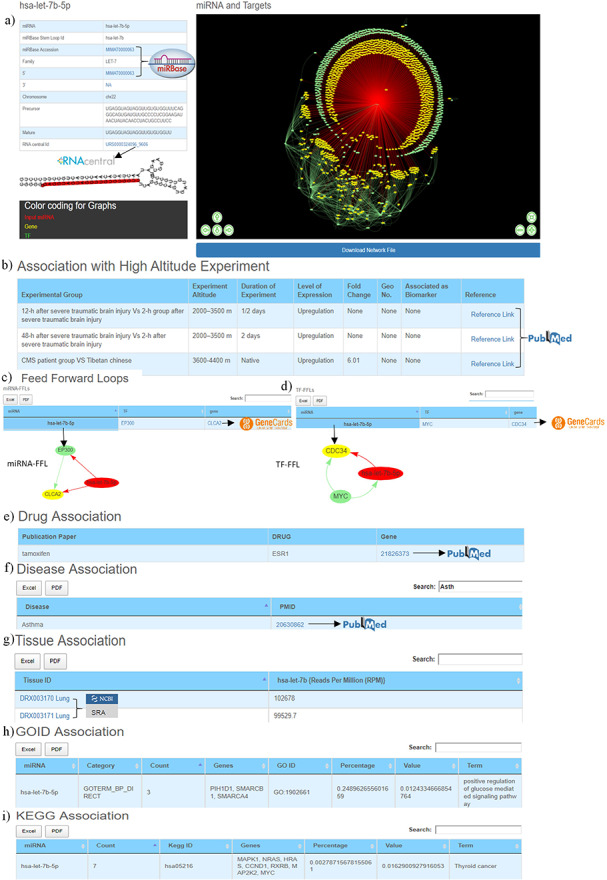
Web image of the miRNA information page. (a) The first section of hsa-let-7b-5p miRNA information page with details about the miRNA and hyperlinking to external databases like miRBase and RNAcentral. (b) The second section shows information related to experimental details of DE human miRNA under HA conditions in published literature. (c) The web image of the miRNA–FFL in tabular format. (d) The web image of the TF–FFL in tabular format. (e) miRNA associations with drug. (f) miRNA associations with different disease. (g) miRNA expression level in different tissues. (h) GO functional enrichment of the miRNA targets. (i) KEGG pathway enrichment of the miRNA targets. The lengthy tables can be searched through ‘search’ box and information in table can be download in both Excel and PDF formats.

The first section of the database provides general information about miRNA such as miRBase accession number, miRBase stem-loop ID, 5ʹ/3ʹ ID, miRNA family, chromosome number, precursor and mature sequences, RNAcentral ID. It also contains miRNA stem-loop structure where mature miRNA sequence is highlighted with red colour (Figure 3a). The miRBase accession, 5ʹ/3ʹIDs and RNA central IDs are also hyperlinked to miRBase and RNAcentral databases to provide additional details of the miRNA like mature and precursor miRNA sequence, miRNA stem-loop structure, chromosome location, source organism, target proteins, target lncRNAs, genome locations, Rfam classification (31, 33).

HAHmiR.DB uniquely offers directed miRNA–TF–gene coregulatory network of the miRNA. The details of the in-house scripts to build this network are provided in the ‘Material and methods’ section. The network nodes are colour coded (miRNA—red, TF—green, target gene—yellow) and interactive with option likes zoom-in/-out, translation of nodes. These networks can be saved in publication quality images. The user can also download the network file, which can also be exported and visualized in publically freely available software’s like Cytoscape (51), BINA (52), Gephi.

### Association of miRNA with HA

For each miRNA, its association with HA is compiled in the form of experimental group, experiment altitude, duration of experiment, level of expression, fold change, GEO accession, association as biomarker and the respective reference, which is hyperlinked to PubMed (53) database that allows easy access to the original publication (Figure 3b).

### Feed-Forward Loops

Biological systems are often built of regulatory circuits that carry out key functions (54). These recurring patterns are very common in gene regulatory networks and are known as network motifs. Important regulatory molecules such as miRNA and TFs often follows these recurring pattern in coregulatory networks to control the complex molecular and cellular responses of living cells (55). FFLs are most overrepresented motifs that are found in the coregulatory networks (56). They are of two types: miRNA–FFLs and TF–FFLs based on the interaction between miRNA and TF. If the interaction is of the type that an miRNA dysregulates TF and both together regulate a target gene, then it is called an miRNA–FFL. A type where a TF regulates an miRNA and both regulate a target gene, then it is called as TF-FFL.

Both these regulatory circuits/FFLs are extracted from miRNA–TF–gene coregulatory networks using in-house scripts as discussed in the ‘Material and methods’ section. The FFLs are provided in a tabular format (Figure 3c, 3d), which can be explored and downloaded. Clicking on the miRNA would provide a network of the FFL in a pop-up window. Each TF and TG symbol in the FFL table is further hyperlinked to the GeneCards (57) database. This would serve as a ready-reference for the user to get additional details about the gene that includes aliases of genes, promoter and enhancer location of gene, protein coded by gene, functional characterization, cellular localization, pathway enrichment, gene–gene interaction network, drug–gene relationship, tissue-specific gene expression profile, orthologs, paralogs, transcript variant, etc.

These regulatory circuits/FFLs could offer mechanistic insights into complex cellular responses and also open new horizons in HA research for identifying potential candidate markers for HA acclimatization.

### Association of miRNA with drugs, diseases and tissues

This section provides details of the drug, disease and tissue association of the miRNAs. The information is represented in three tables. The first table shows information about miRNA and its associated drug compiled from PharmocomiR (Figure 3e). A direct link to the corresponding reference in PubMed is provided for ready-reference. This information may help the user to design/validate miRNA-based drug-targeting/-repurposing experiments. Similarly, miRNA–disease associations (Figure 3f) and miRNA tissue–specific-expression (Figure 3g) are also provided in tabular formats. Each entry in tissue-specific expression table have been hyperlinked to the Sequence Read Archive database (58), which provides additional tissue-specific experimental details (Figure 3g).This could be important to design experiments that aim to study tissue-specific expression. These tables are equipped with the integrated ‘search’ option, and tables can also be downloaded in Excel/PDF format.

### Association of miRNA with GO and KEGG pathways

This section presents the functional characterization of the miRNA gene targets. Both functional and pathway enrichments are stored and visualized in a tabular format. These tables are provided with a ‘search’ option that allows exploring the extensive tables and fetching the user-defined information (gene/GO term/KEGG pathway) easily. Figure 3h shows the GO table searched using the ‘calcium’ keyword to identify specific GO term from the 203 GO entries of hsa-let-7b-5p. Similarly, Figure 3i the ‘PI3K’ keyword was used to search specific entry from the KEGG pathway entries of hsa-let-7b-5p.

**Figure 4.**
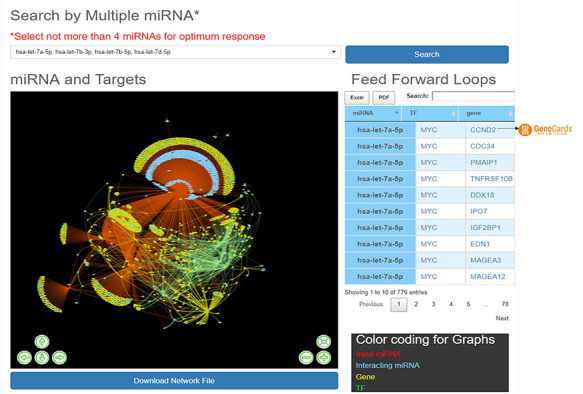
‘Explore’ option of the database. The web image of the section shows complex network of four miRNA, i.e hsa-let-7b-5p, hsa-let-7a-5p, hsa-let-7b-3p, hsa-let-7d-5p and identified FFLs in the adjacent table.

HAHmiR.DB server also allows the user to perform integrated network analysis through the ‘Explore’ option of the database. In this option, the user can select multiple (upto maximum of 4) miRNAs from the database. The server then builds a dynamic miRNA–TF–gene coregulatory network between these miRNAs. The network includes miRNAs, their targeted TFs and genes, and also its interacting miRNAs. The network is presented as an interactive visualization with colour coding and zoom-in and translation options. (Figure 4). The network image may be saved as publication quality images and can also be downloaded. This file can then be used to visualize networks offline in visualization softwares like cytoscape (51), BINA (52), Gephi. HAHmiR.DB also extracts the FFLs in the complex network and presents them in a tabular format. The user can click on each miRNA in the row to see its tripartite graph as pop-ups. The genes and TFs are hyperlinked to GeneCards database. The integrated search option is provided with the table for searching the FFLs using TF/Gene. The FFL table can be downloaded in Excel and PDF formats.

## HAHmiR.DB statistics

HAHmiR.DB contains 556 associations of 384 human miRNAs that are differentially expressed at HA. In terms of interactions, HAHmiR.DB consist of ∼56 000 miRNA target interactions (MTIs) that can be further divided into 38 496 miRNAs→gene, 17 974 miRNA→TF interactions. Additionally, the database contains 47 115 TF→gene and 1500 miRNA→TF interactions. The database also contains 449 unique miRNA–disease associations and 77 miRNA–drug associations. In the database, 52% miRNAs are downregulated and 48% are upregulated. The 556 miRNA entries can also be divided into three categories based on the duration of experiment: 43% of studies had the duration of experiment ranging in months, 40% of studies ranging in days and rest 17% were studies related to HA natives.

**Figure 5.**
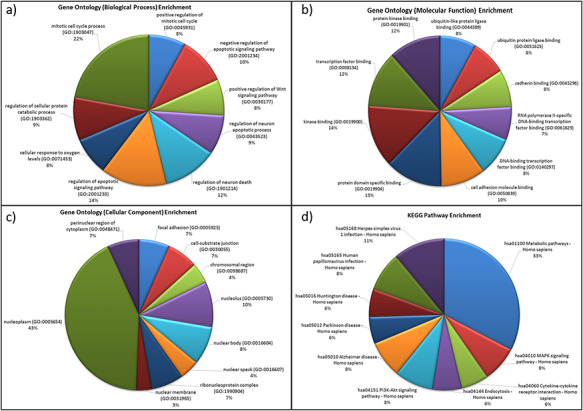
Functional characterization of differentially expressed HA miRNA–targets (a) GO: biological processes. (b) GO: molecular functions (c) GO: cellular compartment and (d) KEGG pathway enrichment.

For the genes in the database, two different types of functional characterizations were performed, i.e GO enrichment and KEGG pathway enrichment. The GO enrichment shows the ‘mitotic cell cycle regulation’, ‘regulation of apoptosis process’, ‘cellular response to oxygen levels’, etc. as the top biological processes (Figure 5a). ‘Cell cycle regulation’ is an important cellular mechanism induced by the hypoxic stress that governs the cell fate from cell proliferation to apoptosis and is widely reported and studied biological process during hypoxic environments (59). The ‘cellular response to oxygen level’ is governed by PHDs oxygen sensors of cells that control the activation of HIF under low-oxygen condition that further regulates hypoxic stress-adaptive responses (7, 59–61). Similarly, ‘transcription factor binding’, ‘protein kinase binding’, ‘cell adhesion molecule binding’, etc. and ‘nucleoplasm (nucleus and cytoplasm)’, ‘nucleolus’, etc. are the top molecular functions and cellular components, respectively (Figures 5b and 5c). These pathways have been reported as hallmark responses to HA stress. Literature shows that protein kinases present in both nucleus and cytoplasm are signal transducers that play important roles in transducing hypoxic stress signals from the cytoplasm to the nucleus for the activation of hypoxic stress-responsive TFs like HIF1, NFKB1 p53, etc. (59). Also, the downregulation of cell adhesion proteins like PECAM-1, ZO-1 during hypobaric hypoxia has been correlated as a signal for vascular leakage and HAPE (62).

The KEGG pathway enrichment shows ‘metabolic pathway’ as most enriched in the miRNA–targets (Figure 5d). Metabolic pathways are responsible in maintaining body-weight homeostasis during HA adaptation/acclimatiz-ation (63).

The functional annotation of miRNA target genes in database shows the correlations of biological processes, molecular functions and pathways with hallmark responses to hypobaric-hypoxic stress and other HA stress conditions (7, 59–61). Hence, this supports the comprehensive biomolecular structure of the database. Thus, HAHmiR.DB is a comprehensive, user-friendly repository of human miRNAs, TFs and genes to study HA stress responses at the molecular level.

At HA, hypobaric hypoxia plays a critical role in triggering an acclimatization mechanism both at the physiological and molecular levels where the lack of oxygen (hypoxia) is sensed by the oxygen sensor of cells and activate master regulators that further control the signalling machinery responsible for hypoxia-adaptive responses in the body. Our database provides the representation of the miRNAs differentially expressed under hypobaric-hypoxic (HA) stress only. Other than hypobaric hypoxia at HA, miRNAs are also expressed in other hypoxic disorders or pathophysiological conditions like stroke, cardiovascular disorders, wounds, malignancies etc. However, their expression level in different hypoxic conditions would be different. Hence, HAHmiR.DB provides molecular changes and expression changes only at HA and not in any generic or other hypoxic microenvironments and hence is representative of molecular responses to HA stress.

## Discussion

HAHmiR.DB is an interactive resource for HA-associated Human miRNAs. The collection has been stored in the MongoDB database that can be updated from time to time. JSON helps in easy and smooth transferring of the data between the server and API. Currently, there is only a single database for HA species genome data set—‘The Yak Genome Database’ that provides information on genomic sequence related of Yak (HA resident animal species) (64). HAHmiR.DB is a unique database that has a collection of DE, HA-specific, human miRNAs that are fetched and manually curated from the literature. The expression profile of an miRNA from different publications is compiled at one place so that it can be compared, analysed and retrieved at ease. The portal enables the user to browse miRNAs individually or allows batch retrieval based on different query filters. It also provides information about the association of these miRNAs in other diseases, its tissue-specific expression and its pharmacological relation with other drugs. It identifies MTIs of each miRNA and also constructs directed tripartite miRNA–TF–gene coregulatory network. Subsequently, HAHmiR.DB identifies regulatory circuits/FFLs in each miRNA–TF–gene coregulatory network. The database also performs integrative analysis with user-defined miRNAs and constructs directed miRNA–TF–gene coregulatory networks of input miRNAs. These miRNA–TF–gene coregulatory networks contain regular miRNA–gene, miRNA–TF, TF–gene and TF–miRNA interactions and additional miRNA–miRNA interactions. This complex network would help understand the biological mechanisms underlining complex interactions. The database is the first database that provides miRNA–TF–gene coregulatory networks and its regulatory circuits in a pathophysiological stress condition.

In this way, HAHmiR.DB helps examine the complex regulation and fine-tuning of the molecular signaling during ascent to HA. These complex alterations decide the fate of an individual to be acclimatized or unacclimatized to HA stress. If unacclimatized, it can result in serious consequences like AMS, CMS, HAPE or HACE. HAHmiR.DB provides a unique platform to perform both a meta-analysis and systematic review of the human HA miRNA studies, compare the results, analyse user-data and also uniquely offer complex regulatory networks. This data could be meaningfully utilized to methodologically study various molecular changes initiated at HA stress and understand the molecular pathways and help identify crucial signalling molecules that could be potential diagnostic, prognostic or therapeutic markers. Regulatory biomolecules like TFs and miRNAs are becoming more helpful in understanding the complex regulation and fine-tuning mechanism in multifactorial disorders and complex pathophysiological conditions like myocardial infarct, cancer, hypoxia (16, 17, 22, 55, 59). Several real-life cases require these molecular markers for predicting individual-acclimatization to HA; for development of molecular remedies to support early acclimatization; improving endurance at HA and improving recovery time; for developing strategies for rapid-induction to HA; for identifying regulatory makeup of HA inhabitant population vs. low landers. Recent literature show that many HA illnesses like AMS, CMS, HAPE, HACE are comorbid to other diseases like obesity, diabetes mellitus, COPD. The molecular cross-connections are however not evidently known. HAHmiR.DB helps in studying molecular mechanisms responsible for acclimatization in HA stress, which could further help to uncover molecular interconnections with comorbid diseases and designing appropriate therapies.

## Supplementary Material

baaa101_Supp
